# Temporal sequencing of symptom change in youth receiving treatment for posttraumatic stress disorder and substance use: secondary findings from a randomised controlled trial

**DOI:** 10.1080/20008066.2026.2630609

**Published:** 2026-03-06

**Authors:** Olivia Dobson, Natalie Peach, Joanne Cassar, Ashling Isik, Louise Bezzina, Olivia Schollar-Root, Ivana Kihas, Katherine A. Dobinson, Vanessa E. Cobham, Emma L. Barrett, Sean Perrin, Sarah Bendall, Sudie E. Back, Kathleen Brady, Bronwyn Milne, Maree Teesson, Katherine L. Mills

**Affiliations:** a The Matilda Centre for Research in Mental Health and Substance Use, The University of Sydney, Sydney, Australia; b School of Psychology, University of Queensland, Brisbane, Australia; c Department of Psychology, Lund University, Lund, Sweden; d Orygen National Centre of Excellence in Youth Mental Health, Centre for Youth Mental Health, Parkville, University of Melbourne, Australia; e Department of Psychiatry and Behavioral Sciences, Medical University of South Carolina, Charleston, SC, USA; f Department of Adolescent Medicine, The Sydney Children's Hospitals Network, Sydney, Australia

**Keywords:** Posttraumatic stress disorder, substance use disorder, adolescents and young adults, youth, symptom severity, randomised clinical trials, Trastorno de estrés postraumático, trastorno por uso de sustancias, adolescentes y adultos jóvenes, jóvenes, gravedad de los síntomas, ensayos clínicos aleatorizados

## Abstract

**Background:**

Posttraumatic stress disorder (PTSD) and substance use disorders (SUD) frequently co-occur in youth. Integrated, trauma-focused treatments are recommended, but evidence in youth is limited and the dynamics of symptom change are poorly understood.

**Objectives:**

We investigated within-treatment changes in PTSD and substance use, and their temporal sequencing, in a randomised controlled trial (RCT) comparing integrated treatment with supportive counselling among youth aged 13–25 years.

**Method:**

Participants (*n* = 55) were randomised to an integrated, exposure-based treatment, Concurrent Treatment of PTSD and SUD Using Prolonged Exposure – Adolescent version (COPE-A); or supportive counselling, Person-Centred Therapy (PCT). PTSD and substance use symptoms were assessed at each session. Generalised estimating equations were used to analyse symptom change over time, and Spearman’s correlations were used to examine associations between early (sessions 1-5) and later (sessions 5-11) change.

**Results:**

COPE-A showed significantly greater reductions in total PTSD symptom severity during treatment (*β* = −17.00, 95% CI −29.50 to −4.46); with no between-group differences in substance use quantity or frequency. PTSD symptom clusters improved concurrently, but no temporal relationships were observed. Temporal relationships for substance use change were limited, and changes in PTSD symptoms were not associated with concurrent or subsequent changes in substance use.

**Conclusion:**

Integrated, trauma-focused treatment reduced PTSD symptoms, reinforcing the safety and tolerability of exposure-based therapy in this youth sample with comorbid PTSD-SUD. PTSD improvement was not associated with either concurrent or subsequent reductions in substance use. Understanding temporal patterns of change may help refine models of PTSD-SUD comorbidity and optimise intervention delivery.

## HIGHLIGHTS

- This is the first randomised trial to examine how posttraumatic stress and substance use symptoms change over time in youth with co-occurring posttraumatic stress disorder (PTSD) and substance use disorders (SUD).
- An integrated, exposure-based treatment (Concurrent Treatment of PTSD and SUD Using Prolonged Exposure – Adolescent Version; COPE-A) produced significantly greater reductions in posttraumatic stress symptoms than supportive counselling.
- Findings suggest that COPE-A is a safe and effective approach for youth with PTSD-SUD, however neither concurrent nor temporal relationships were observed between changes in PTSD and SUD symptoms, offering new insight into treatment mechanisms and ways to refine integrated care.

## Abstract (es)

**Antecedentes:** El trastorno de estrés postraumático (TEPT) y el trastorno por uso de sustancias (TUS) con frecuencia co-ocurren en jóvenes. Se recomiendan los tratamientos integrados centrados en trauma, pero la evidencia en jóvenes es limitada y las dinámicas del cambio de síntomas es poco conocida.

**Objetivos:** Investigamos, los cambios dentro del tratamiento en TEPT y consumo de sustancias y su secuencia temporal, en un ensayo controlado aleatorizado (ECA) comparando el tratamiento integrado con consejería de apoyo entre jóvenes de 13 a 25 años.

**Método:** Los participantes (*n* = 55) fueron asignados aleatoriamente a un tratamiento integrado basado en exposición, el Tratamiento Concurrente del TEPT y TUS Utilizando Exposición Prolongada versión para Adolescentes (COPE-A; o a consejería de apoyo, Terapia Centrada en la Persona (TCP). Los síntomas de TEPT y consumo de sustancias se evaluaron en cada sesión. Se utilizaron ecuaciones de estimación generalizadas para analizar el cambio de los síntomas a lo largo del tiempo las correlaciones de Spearman para examinar las asociaciones entre los cambios tempranos (sesiones 1-5) y posteriores (sesiones 5-11).

**Resultados:** COPE-A mostró reducciones significativamente mayores en la gravedad total de los síntomas del TEPT durante el tratamiento (*β* = −17.00, IC 95% −29.50 a −4.46; sin diferencias intergrupales en la cantidad ni frecuencia del consumo de sustancias. Los grupos de síntomas del TEPT mejoraron simultáneamente, pero no se observaron relaciones temporales. Las relaciones temporales para el cambio del consumo de sustancias fueron limitadas y los cambios en los síntomas de TEPT no se asociaron con cambios simultáneos o posteriores en el consumo de sustancias. Conclusión: El tratamiento integrado centrado en el trauma redujo los síntomas de TEPT, lo que refuerza la seguridad y tolerabilidad de la terapia basada en la exposición en esta muestra de jóvenes con TEPT-TUS comórbidos. La mejoría del TEPT no se asoció ni con reducciones simultáneas ni posteriores en el consumo de sustancias. La comprensión de los patrones temporales de cambio puede refinar los modelos de comorbilidad de TEPT-TUS y optimizar la aplicación de la intervención.

## 1. Introduction

Posttraumatic stress disorder (PTSD) and substance use disorders (SUDs) are common disorders that frequently co-occur (Gielen et al., 2012; Robinson & Deane, 2022). Both conditions typically emerge during adolescence and early adulthood, developmental periods marked by initiation of substance use (AIHW, 2025; Patrick et al., 2024) and heightened exposure to traumatic events (Darnell et al., 2019; McLaughlin et al., 2013; Saunders & Adams, 2014). Among youth exposed to trauma, 14-16% meet diagnostic criteria for PTSD (Alisic et al., 2014; Nooner et al., 2012); and up to half develop a comorbid SUD (Nooner et al., 2012; Basedow et al., 2020; Simmons & Suárez, 2016).

The self-medication hypothesis, a leading framework for understanding PTSD-SUD comorbidity, proposes that individuals use substances to cope with trauma-related distress (Hawn et al., 2020). Existing literature provides some support for the self-medication hypothesis as an etiologic model; however, this evidence remains methodologically constrained, due to a predominance of cross-sectional designs, inconsistent operationalisation of trauma-specific coping, and limited capacity to establish temporal precedence between PTSD symptoms and substance use (Hawn et al., 2020). Additionally, a growing body of research suggests that the self-medication hypothesis processes may play a role in the maintenance of PTSD-SUD comorbidity. Across substances, elevations in PTSD symptoms, particularly intrusion, hyperarousal, and negative affect, have been shown to temporally precede increases in craving and, in some studies, subsequent substance use, whereas the reverse temporal sequence is less consistently observed (Renaud et al., 2021). These findings are consistent with patient reports describing increased substance use during periods of PTSD symptom exacerbation (Back et al., 2014; Gielen et al., 2016; Mefodeva et al., 2023).

Given the high comorbidity between PTSD and SUD, best practice recommends integrated treatments that address both disorders concurrently (Hien et al., 2024). Several integrated interventions have demonstrated efficacy in reducing both PTSD and SUD symptoms (Back et al., 2012; Brady et al., 2001; Hien et al., 2009; McGovern et al., 2009; Mills et al., 2012) however, exposure-based approaches have shown greater PTSD symptom reduction than those primarily targeting substance use (Hien et al., 2024). With few recent exceptions (Danielson et al., 2020); this evidence comes almost entirely from adult populations. Because comorbid PTSD-SUD is linked to substantial symptom burden and functional impairment across education, employment, and health that can persist into adulthood (Barrett et al., 2014; Blanco et al., 2013; Norman et al., 2018; Ouimette et al., 2006; Rodriguez et al., 2019; Stein et al., 2017); there is a critical need to investigate the effectiveness of integrated treatments in youth to inform early intervention efforts (Hall et al., 2016).

Understanding mechanisms of change in PTSD-SUD is essential to refine treatment approaches. It remains unclear whether the self-medication hypothesis continues to explain symptom interplay during treatment. Examining trajectories of both conditions within treatment, rather than pre–post change alone, can help clarify mechanisms and guide targeted care (Kazdin, 2009; Laurenceau et al., 2007). Clarifying when and how changes occur may help elucidate the temporal ordering of symptom change across domains, including whether improvements occur concurrently or sequentially.

A small body of adult research has examined the temporal sequencing of symptom change in PTSD-SUD within-treatment. Some studies suggest that reductions in PTSD symptoms precede and are associated with decreases in substance use (Back et al., 2006; Hien et al., 2025, 2010; Tripp et al., 2020) and that changes in the SUD symptoms are mediated by change in PTSD symptom severity (Hien et al., 2025; Ruglass et al., 2025); which may support the self-medication hypothesis. However, these findings are equivocal and other research reports no consistent directionality between symptom changes (Badour et al., 2022; Peirce et al., 2020). Few studies have assessed change at the level of PTSD symptom clusters, aside from early adult trials incorporating medication (Back et al., 2006); which may provide important insights into mechanisms of change. Importantly, almost all available evidence derives from adult populations. The current study aimed to address this gap by examining changes in PTSD symptoms and substance use among youth enrolled in a randomised controlled trial (RCT) evaluating an integrated treatment for PTSD-SUD versus non-directive supportive therapy (Peirce et al., 2020). While the primary RCT evaluates treatment efficacy using baseline, post-treatment, and follow-up assessments, the present secondary analysis leverages session-by-session data collected during treatment to examine within-treatment symptom trajectories and temporal sequencing. This approach allows investigation of change processes that cannot be assessed using pre–post or follow-up designs alone. We sought to (1) examine changes in PTSD severity and substance use (quantity and frequency) across key treatment phases, compared with a control group; and (2) explore the temporal sequencing of symptom change across PTSD and substance use domains. As this is the first study to investigate within-treatment trajectories and their interrelationships in youth receiving integrated exposure-based therapy, analyses were considered exploratory.

## 2. Methods

### 2.1. Participants

Between June 2018 and June 2022, participants were recruited across Greater Sydney via clinical services, community referrals, and targeted advertising. Inclusion criteria were: past-month alcohol or other drug use; score ≥ 2 on the Car; Relax; Alone; Forget; Friends; Trouble Questionnaire (CRAFFT) (Knight et al., 1999) indicating a history of problematic alcohol or other drug use; lifetime exposure to at least one traumatic event (UCLA PTSD Reaction Index (UCLA PTSD-RI) (Steinberg et al., 2013)) and a past-month Diagnostic and Statistical Manual of Mental Disorders, 5th Edition (DSM-5) PTSD diagnosis or subthreshold PTSD (defined as meeting criteria A, F and G and at least one symptom across B–E); and fluency in English. Furthermore, participants aged 12–25 were eligible for inclusion in this study, although the final sample included participants aged between 13–25 (Table 1).

**Table 1.** Sample characteristics

Characteristic	COPE-A (*n* = 24)	PCT (*n* = 25)	Total (*n* = 49)
*Demographics*			
Mean age in years (*SD*)	19.4 (3.2)	19.6 (3.0)	19.5 (3.2)
Identifying as female, *n* (%)	16 (66.7)	18 (72.0)	34 (69.4)
*Sexuality*			
Heterosexual, *n* (%)	15 (62.5)	13 (52.0)	28 (57.1)
Gay/lesbian/homosexual, *n* (%)	1 (4.2)	3 (12.0)	4 (8.2)
Bisexual, *n* (%)	4 (16.7)	7 (28.0)	11 (22.4)
Other, *n* (%)	4 (16.7)	2 (8.0)	6 (12.2)
Australian born (%)	22 (91.7)	23 (92.0)	45 (91.8)
Currently attending school or tertiary education, *n* (%)	15 (62.5)	19 (76.0)	34 (69.4)
Ever suspended, n (%)	14 (60.9)	11 (44.0)	25 (52.1)
Ever expelled, *n* (%)	2 (8.7)	1 (4.0)	3 (6.3)
Ever arrested, *n* (%)	8 (33.3)	6 (24.0)	14 (28.6)
*Current accommodation*			
Parents’ home, *n* (%)	8 (33.3)	12 (48.0)	20 (40.8)
Other family home, *n* (%)	1 (4.2)	2 (8.0)	3 (6.1)
Own house or flat, *n* (%)	12 (50.0)	11 (44.0)	23 (46.9)
Shelter/refuge, *n* (%)	3 (12.5)	0 (0.0)	3 (6.1)
Ever homeless, *n* (%)	11 (45.8)	7 (28.0)	18 (36.7)
Juvenile detention history, *n* (%)	3 (12.5)	1 (4.0)	4 (8.2)
Prison history, *n* (%)	1 (4.2)	0 (0.0)	1 (2.0)
*Substance use characteristics*			
Age at first use, mean (*SD*)	12.1 (2.9)	12.8 (3.2)	12.4 (3.0)
*Drug of choice*			
Cannabis	12 (50.0)	13 (52.0)	25 (51.0)
Alcohol	12 (50.0)	12 (48.0)	24 (49.0)
No. of SUD criteria met on DISC, median (range)	8.1 (2.6)	8.2 (2.0)	8.2 (2.3)
Diagnosis of SUD, *n* (%)	23 (95.83)	25 (100.0)	54 (98.1)
*SUD severity (DISC; n (%))*			
Mild or below	2 (8.4)	1 (4.0)	3 (6.1)
Moderate	1 (4.2)	1 (4.0)	2 (4.1)
Severe	21 (87.5)	23 (92.0)	44 (89.8)
*No. of drug types used, median (range)*			
Past month	4.0 (1-9)	5.0 (2-9)	4.0 (1-11)
Lifetime	10.0 (5-17)	10.0 (2-15)	10.0 (2-17)
No. of days used any substance in past month, mean (*SD*)	18.3 (9.3)	19.4 (9.7)	18.9 (9.4)
*Trauma characteristics*			
Simple/single incident, *n (%)*	0 (0.0)	1 (4.0)	1 (2.0)
Complex/multiple incident, *n (%)*	24 (100.0)	24 (96.0)	48 (98.0)
No. of trauma types experienced, median (range)	6.0 (2-11)	5.0 (1-9)	6.0 (1-11)
Age of first trauma exposure, mean (*SD*)	7.4 (4.2)	6.3 (4.3)	6.9 (4.3)
Age at index trauma exposure, mean (*SD*)	11.6 (6.0)	10.1 (6.5)	10.8 (6.3)
PTSD diagnosis (CAPS-CA-5; *n (%))*			
Full diagnosis	23 (95.8)	20 (80.0)	43 (87.8)
Subthreshold diagnosis	0 (0.0)	2 (8.0)	2 (4.1)
Dissociative subtype	10 (41.7)	9 (36.0)	19 (38.8)
*PTSD symptom severity (CAPS-CA-5; mean (SD))*			
Total	35.8 (9.6)	35.9 (9.7)	35.9 (9.6)
Intrusions	9.0 (3.6)	8.5 (3.4)	8.7 (3.5)
Avoidance	4.3 (1.3)	3.4 (1.5)	3.9 (1.5)
Negative cognitions and mood	13.5 (3.3)	14.0 (4.9)	13.8 (4.2)
Hyperarousal	9.0 (3.5)	10.0 (4.0)	9.5 (3.8)
Duration of symptoms in years, median (range)	3.0 (0.2-21.0)	2.0 (0.1-22.0)	2.0 (0.1-22.0)

Note. COPE-A = Concurrent Treatment of PTSD and SUD Using Prolonged Exposure, Adolescent version; PCT = Person-Centred Therapy; DISC = Diagnostic Interview Schedule for Children; PTSD = posttraumatic stress disorder; CAPS-CA-5 = Clinician-Administered PTSD Scale for Children and Adolescents for DSM-5.

Participants were excluded if they had a recent history of attempted suicide (i.e. within the past 3 months) or current imminent risk of suicide or serious self-harm, current symptoms of psychosis based on the Mini International Neuropsychiatric Interview for Children and Adolescents (MINI-KID) (Sheehan et al., 2010) score of >1 and clinical observation; cognitive impairment severe enough to impede treatment based on clinical observation, and ongoing trauma-related threat or ongoing unsupervised contact with the alleged perpetrator (in cases where participants experienced a traumatic event involving interpersonal violence or abuse).

Fifty-five participants were randomised, and those who commenced therapy were included in the current analysis (*n* = 49). Intention-to-treat analyses for primary and secondary outcomes are reported elsewhere (Mills et al. ). Written informed consent was obtained from all participants. Parent or caregiver consent was sought for those under 16 years of age; however, for those aged 14–15 years who were deemed to be mature minors, the young person’s consent alone was accepted if parental consent was not possible (e.g. parents unwilling to engage). The study was approved by the Human Research Ethics Committees of the Sydney Children’s Hospital Network (HREC/17/SCHN/306) and the University of Sydney (2018/863). The trial was preregistered at the Australia New Zealand Clinical Trials Registry (ACTRN12618000785202).

### 2.2. Interventions

Intervention allocation was conducted independently by the NHMRC Clinical Trials Centre after the baseline interview using a process of minimisation to ensure balance by sex, age, SUD severity, trauma type, and PTSD symptom severity. Both conditions involved up to 16 individual 60–90-minute sessions.

#### 2.2.1. Concurrent Treatment of PTSD and SUD Using Prolonged Exposure - Adolescent version (COPE-A)

The COPE-A intervention is a developmentally adapted, integrated treatment targeting PTSD-SUD, grounded in prolonged exposure and cognitive–behavioural therapy (CBT). COPE-A incorporates core elements of trauma-focused CBT (including imaginal and in vivo exposure); CBT for substance use, psychoeducation, and motivational interviewing, with developmentally-appropriate modifications (e.g. simplified rationales, age-relevant examples, and enhanced focus on engagement, and emotional regulation) (Mills et al., 2020). Initial sessions focused on risk management, psychoeducation, and motivational enhancement. Prolonged exposure commenced at sessions five, and was delivered alongside CBT skills for distress tolerance, craving management, and interpersonal communication (Schollar-Root et al., 2022).

#### 2.2.2. Person-Centred Therapy (PCT).

The comparison condition was manualised PCT, an active, non-directive intervention rooted in core principles of therapeutic change: genuineness, unconditional positive regard, and accurate empathy. Therapists provided empathic listening and support while participants led the content of sessions. This condition reflected common clinical practice while ensuring consistency in delivery. PCT has demonstrated effectiveness in prior trials targeting PTSD-SUD (Ehlers et al., 2010; Kay-Lambkin et al., 2011).

### 2.3. Measures

Participants completed structured interviews at baseline assessing demographic characteristics, trauma exposure, PTSD symptoms, and substance use. Interviews included the Adverse Childhood Experiences (ACE) Questionnaire (Dube et al., 2001); DSM-5 version of the clinician-administered UCLA PTSD Reaction Index (PTSD-RI) (Steinberg et al., 2013); Clinician-Administered PTSD Scale for Children and Adolescents for DSM-5 (CAPS-CA-5) (Nader et al., 2013); Timeline Followback (TLFB) (Sobell & Sobell, 1992); and the Diagnostic Interview Schedule for Children (DISC) (Shaffer et al., 2000). Additional details are reported elsewhere (Mills et al. Mills et al., 2020).

The PTSD-RI and TLFB were administered at the beginning of each session to assess within-treatment changes in PTSD symptoms and the frequency and quantity of use of each participant’s primary substance of concern. Alcohol or cannabis were the primary substances of concern for all participants. Frequency and quantity reported reflect data pertaining only to the primary substance and did not reflect use of other substances. Standard alcohol units were calculated using Australian guidelines (National Health and Medical Research Council 2020). Australian Guidelines to Reduce Health Risks from Drinking Alcohol [Internet]; and cannabis use was converted to estimated THC-equivalent standard cannabis units following established guidelines (Freeman & Lorenzetti, 2020). Cannabis quantities were standardised across different delivery methods using published THC estimates from Australian or comparable international samples (e.g. grams (Swift et al., 2013); joints, tubes, and cones (Dawson et al., 2024); edibles (Callaghan et al., 2019)). To create a comparable composite quantity measure, individual quantity scores were standardised (z-scores); retaining only scores related to each participant's main drug of concern. This variable was transformed by adding two to remove negative values and log-transformed to correct for skewness.

### 2.4. Statistical analysis

**Figure 1.**
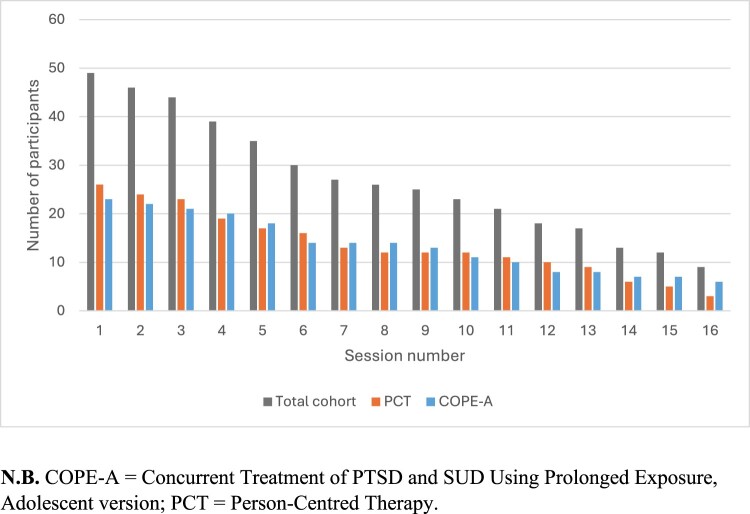
Participant attendance at each session by treatment group.

Generalised estimating equations (GEE) were used to model within- and between-group changes in PTSD severity and substance use (quantity and frequency) in R version 4.5.2. Participant attendance over the sixteen sessions is presented in Figure 1 for the total cohort, COPE-A and PCT groups. Given attrition over sessions, between-session comparisons were limited to Session 1 (S1); Session 5 (S5); marking the last measurement time point before the commencement of prolonged exposure; and Session 11 (S11; the last session with sufficient sample size to enable comparisons: A minimum of n ≥ 10 per condition to avoid unstable estimates at later sessions with sparse data). To account for the small number of clusters, GEE’s with a bias-adjusted covariance estimator were used (Mancl & DeRouen, 2001).

Linear GEE models were applied to continuous outcomes, including PTSD total scores, PTSD symptom cluster scores, and substance use quantity, with results reported as unstandardised mean differences and 95% confidence intervals (CIs). Substance use frequency was analysed as a count outcome using a Poisson GEE with log link, and results are reported as incidence rate ratios (IRRs) with 95% CIs*.*

All models specified an exchangeable working correlation structure to account for within-subject correlations over time. Predictor terms included treatment group (COPE-A vs PCT); time (S1, S5, S11); and group × time interaction, to evaluate differential change over time by treatment condition. Where group × time interactions were non-significant, the interaction effect was removed from the model and main effects interpreted.

Investigation of the temporal sequencing of PTSD and substance use (quantity and frequency) change was conducted using Spearman’s rank-order correlations using IBM SPSS Statistics (Version 28.0.0.0). Because Aim 2 focused on examining temporal relationships between symptom changes as indicators of within-treatment processes, analyses were conducted on the combined sample; treatment group–stratified sensitivity analyses were performed to assess whether these relationships differed by intervention. Correlations were calculated on change scores computed separately for the early (S1-S5) and later (S5 to S11) treatment phases. Methodological work has highlighted that difference-score models remain appropriate for studying change when more complex longitudinal models are underpowered (Castro-Schilo & Grimm, 2018). Analyses examined concurrent associations (changes occurring within the same treatment phase) and temporal associations (changes between early and later phases). Confidence intervals (95%) were estimated via bootstrapping with 1,000 resamples using the percentile method.

## 3. Results

### 3.1. Baseline sample characteristics

The characteristics of the sample included in these analyses (*n* = 49; those who attended ≥ 1 session) are similar to those observed in the full sample (*n* = 55) (Peach et al., 2024); with few between group differences (Table 1). Participants’ mean age was 19.5 years (*SD* = 3.2, range = 13-25 years). The most reported drug of choice at baseline was cannabis (51.0%); followed by alcohol (49.0%). Participants reported a median of 4.0 (range 1-11) drugs used in the past month, most commonly alcohol and cannabis. Nearly all participants had experienced multiple traumas (median = 6.0; range = 1-11). Most participants (87.8%) met full-threshold DSM-5 PTSD diagnostic cut-offs, with an average CAPS-CA-5 total score of 35.9 (*SD* = 9.6). Further details on sample characteristics are available elsewhere (Peach et al., 2024)**.**

### 3.2. Within-treatment changes

#### 3.2.1. PTSD symptom severity

**Figure 2.**
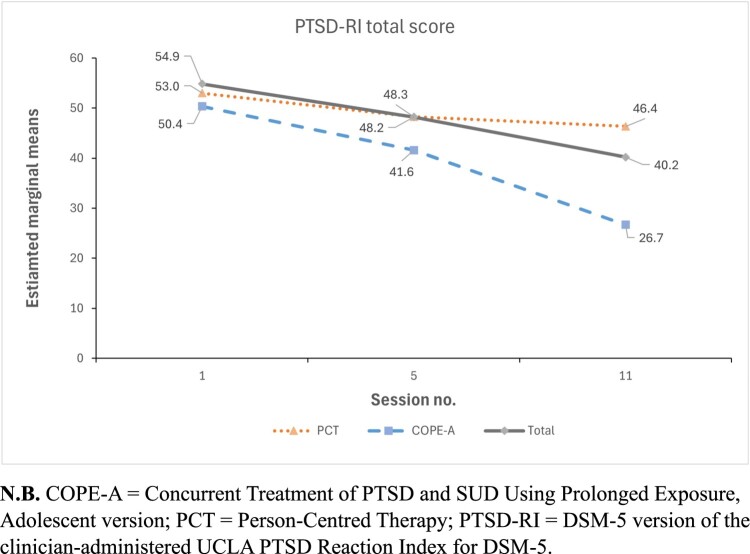
Within-treatment trajectories of PTSD symptoms across S1, S5, and S11 for COPE-A, PCT and total cohort groups. Fitted estimates based on generalised estimating equation models. PTSD-RI scores (84)*.*

The results of the GEE examining within-treatment changes in PTSD outcomes are presented in Table 2 and depicted in Figure 2. There was a significant group × time interaction for PTSD symptom severity (χ^2^_1_ = 7.38, *p* = .025). The COPE-A group demonstrated significant reductions in symptoms between S1 and S5 (mean difference, −8.77, *p* = .005) with further reductions observed between S5 and S11 (mean difference, −14.87, *p* = .002). The PCT group did not demonstrate significant change in PTSD symptom severity during either treatment phase. Overall, COPE-A demonstrated a 17.00-point greater reduction in PTSD severity scores relative to PCT between S1 and S11, with the greatest change occurring between S5 and S11 (mean difference, −12.95; *p* = .025).

**Table 2.** Unadjusted comparison between COPE-A and PCT cohorts on PTSD outcomes measured with the PTSD-RI.

		Mean (95% CI)			Mean Difference (95% CI)					
Outcome measure		Session 1	Session 5	Session 11	Within-Group Difference between Session 1 and Session 5	Between-Group Difference between Session 1 and Session 5	Within-Group Difference Between Session 5 and Session 11	Between-Group Difference Between Session 5 and Session 11	Within-Group Difference Between Session 1 and Session 11	Between-Group Difference Between Session 1 and Session 11
**PTSD-RI Total**	COPE-A	50.35 (44.68 to 56.02)	41.58 (33.61 to 49.55)	26.70 (16.22 to 37.08)	−8.77 (−14.99 to −2.55)*^c^*	−4.05 (−13.60 to 5.51)	−14.87 (−24.18 to −5.57)^b^	−12.95 (−24.32 to −1.59)^c^	−23.64 (−32.08 to −15.21)*^a^*	−17.00 (−29.50 to −4.46)^b^
	PCT	53.00 (47.70 to 58.32)	48.28 (39.88 to 56.69)	46.36 (36.36 to 56.37)	−4.72 (−12.00 to 2.55)	1 [Reference]	−1.92 (−8.45 to 4.61)	1 [Reference]	−6.64 (−15.90 to 2.65)	1 [Reference]
	Between-group difference at each interview, Mean (95% CI)	−2.65 (−10.40 to 5.13)	−6.70 (−18.29 to 4.88)	−19.66 (−34.07 to −5.25)^c^						
**Cluster B – Intrusions**	COPE-A	12.26 (10.40 to 14.11)	10.49 (7.78 to 13.19)	5.95 (2.54 to 9.37)	−1.77 (−3.99 to 0.44)	0.06 (−3.02 to 2.91)	−4.53 (−7.88 to −1.18)^c^	−4.35 (−8.23 to −0.46)^c^	−6.31 (−9.26 to −3.36)^a^	−4.29 (−8.19 to −0.39)^c^
** **	PCT	13.04 (11.47 to 14.61)	11.21 (8.97 to 13.45)	11.02 (8.08 to 13.96)	−1.83 (−3.80 to 0.53)	1 [Reference]	−0.19 (−2.14 to 1.76)	1 [Reference]	−2.02 (−4.57 to 0.53)	1 [Reference]
** **	Between-group difference at each interview, Mean (95% CI)	−0.78 (−3.21 to 1.65)	−0.72 (−4.23 to 2.79)	−5.07 (−9.58 to −0.56)^c^						
**Cluster C – Avoidance**	COPE-A	5.52 (4.70 to 6.35)	4.60 (3.53 to 5.67)	2.59 (1.54 to 3.74)	−0.92 (−1.90 to 0.06)	1.20 (−0.13 to 2.53)	−2.01 (−3.13 to −0.89)^a^	−1.61 (−3.08 to −0.13)^c^	−2.93 (−4.03 to −1.82)^a^	−2.81 (−4.41 to −1.21)^a^
** **	PCT	5.23 (4.54 to 5.92)	5.51 (4.51 to 6.51)	5.10 (4.01 to 6.21)	0.28 (−0.62 to 1.18)	1 [Reference]	−0.40 (−1.36 to 0.56)	1 [Reference]	−0.12 (−1.28 to 1.04)	1 [Reference]
** **	Between-group difference at each interview, Mean (95% CI)	0.29 (−0.78 to 1.36)	−0.91 (−2.37 to 0.55)	−2.81 (−4.41 to −1.21)^a^						
**Cluster D – Negative alterations in cognition and mood**	COPE-A	18.78 (16.66 to 20.90)	14.49 (11.62 to 17.35)	9.25 (5.34 to 13.17)	−4.29 (−6.26 to −2.33)^a^	−2.19 (−5.55 to 1.16)	−5.23 (−8.76 to −1.71)^b^	−5.33 (−9.84 to −0.82)^c^	−9.53 (−12.94 to −6.12)^a^	−7.52 (−12.36 to −2.69)^b^
** **	PCT	19.31 (17.10 to 21.52)	17.21 (13.94 to 20.47)	17.30 (13.49 to 21.11)	−2.10 (−4.82 to 0.62)	1 [Reference]	0.09 (−2.71 to 2.90)	1 [Reference]	−2.01 (−5.43 to 1.42)	1 [Reference]
** **	Between-group difference at each interview, Mean (95% CI)	0.53 (−3.59 to 2.54)	−2.72 (−7.06 to 1.62)	−8.05 (−13.51 to −2.58)^b^						
**Cluster E – Alterations in arousal and reactivity**	COPE-A	13.78 (11.88 to 15.68)	12.02 (9.96 to 14.09)	8.94 (6.21 to 11.68)	−1.76 (−3.56 to −0.04)	−0.81 (−3.83 to 2.21)	−3.08 (−5.58 to −0.58)*^c^*	−1.67 (−4.98 to 1.65)	−4.84 (−6.99 to −2.69)*^a^*	−2.47 (−6.38 to 1.24)
** **	PCT	15.42 (13.48 to 17.37)	14.47 (11.88 to 17.06)	13.06 (9.71 to 16.40)	−0.95 (−3.39 to 1.48)	1 [Reference]	−1.41 (−3.58 to 0.76)	1 [Reference]	−2.37 (−5.63 to 0.90)	1 [Reference]
** **	Between-group difference at each interview, Mean (95% CI)	−1.64 (−4.36 to 1.08)	−2.45 (−5.76 to 0.86)	−4.11 (−8.43 to −0.21)						

Note. COPE-A = Concurrent Treatment of PTSD and SUD Using Prolonged Exposure, Adolescent version; PCT = Person-Centred Therapy; PTSD-RI = Posttraumatic Stress Disorder Reaction Index; IRR = incidence rate ratio; CI = confidence interval.

^a^ *p* ≤ .001.

^b^ *p* < .005.

^c^ *p* < .05.

**Figure 3.**
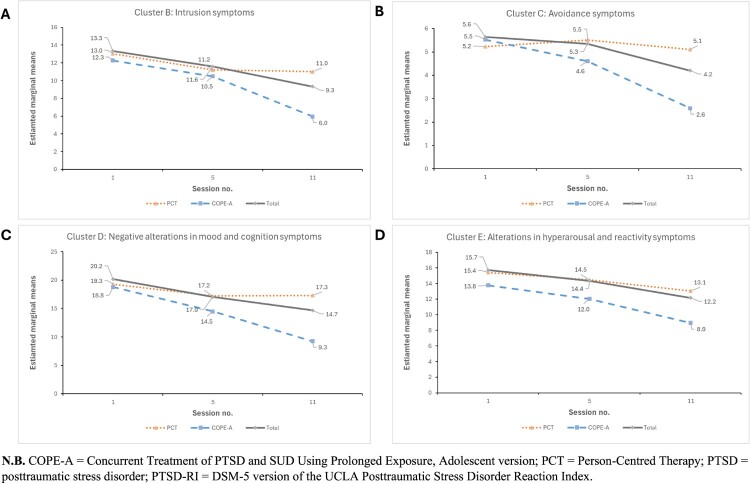
Within-treatment trajectories of DSM-5 PTSD cluster scores across S1, S5, and S11 for COPE-A, PCT and total cohort groups, including (A) Intrusions, (B) Avoidance, (C) Negative alterations in cognition and mood, (D) Alteration in arousal and reactivity measured with the PTSD-RI.

Regarding symptom cluster scores (Figure 3); significant group × time interactions were observed for Cluster B (intrusions) (χ² = 5.54, *p* = .006) and Cluster C (avoidance) (χ^2^_1_ = 11.82, *p* = .002). Neither COPE-A nor PCT demonstrated significant change in Cluster B or Cluster C symptoms from S1 to S5. The COPE-A group, however, showed significant improvements from S5 to S11 (Cluster B: mean difference, −4.53, *p* = .008; Cluster C: mean difference, −2.01, *p* < .001); whereas the PCT group continued to evidence no significant change for either symptom cluster. Overall, from S1 to S11, COPE-A demonstrated a 4.29-point (*p* *=* .03) greater reduction in Cluster B symptoms and a 2.81-point (*p* < .001) greater reduction in Cluster C symptoms relative to PCT.

In relation to Cluster D (negative alterations in cognition and mood); the group × time interaction was significant (χ^2^_1_ = 9.30, *p* = .009). COPE-A demonstrated significant reductions in symptom severity in both treatment phases (S1-S5: mean difference, −4.29, *p* < .001; S5-S11: mean difference, −5.23, *p* = .004); while PCT did not. Overall, COPE-A demonstrated a 7.52-point (*p* = .002) greater reduction in total PTSD severity scores relative to PCT between S1 and S11, with the greatest change occurring between S5 and S11 (mean difference, −5.33, *p* = .021). For Cluster E (alterations in arousal and reactivity) symptoms, no significant group × time interaction was observed (χ^2^_1_ = 1.59, *p* = .452). Only the COPE-A showed within-group improvements in both the overall treatment (S1-S11 mean difference: −4.84, *p* < .001) treatment and the later treatment phase (S5-S11: mean difference, −3.08, *p* = .015). No significant between-group difference was observed relative to the PCT group**.** When the group × time interaction effect was removed from the model, there was a significant effect for time (*χ*^2^_2_ = 12.9, *p* = .002); but not for group (χ^2^_1_ = 2.85, *p* = .091). This indicates that both groups experienced a significant reduction in Cluster E symptoms between S1 and S11 (mean difference, −3.55, *p* < .001); with the greatest change occurring between S5 and S11 (mean difference, −2.20, *p* = .008). No significant between-group differences were observed.

#### 3.2.2. Substance use

**Figure 4.**
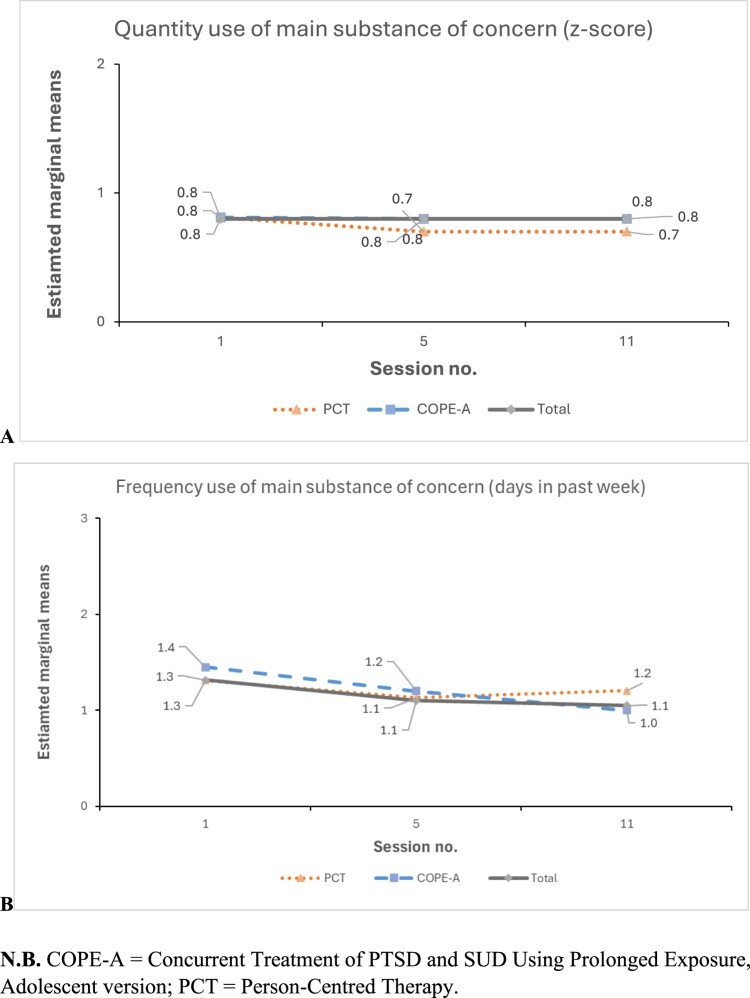
Within-treatment trajectories of substance use across S1, S5, and S11 for COPE-A, PCT and total cohort groups, including (A) quantity use of main substance of concern, and (B) frequency use of main substance of concern.

There was no significant group × time interaction for either quantity (χ^2^_1_ = 1.08, *p* = .582) or frequency (χ^2^_1_ = 1.96, *p* = .376) of substance use (Table 3 and Figure 4). COPE-A demonstrated significant reductions in substance use frequency from S1 to S5 (mean difference, −0.78; *p* = .029) and overall, between S1 and S11 (mean difference, −0.93; *p* = .049); however, no significant changes were observed for substance use quantity. No significant changes were observed for the PCT group across any treatment phase, nor were there significant between-group differences in either frequency or quantity substance use. When the group × time interaction effect was removed from the quantity model, there were no significant effects for either group (χ^2^_1_ = 0.05, *p* = .821) or time (χ^2^_2_ = 1.37, *p* = .505); reflecting no change for either group. When the interaction effect was removed from the frequency model, there was no significant effect for group (χ^2^_1_ = 0.14, *p* = .705); although a significant effect was observed for time (χ^2^_2_ = 10.33, *p* = .006).

**Table 3.** Unadjusted comparison between the COPE-A and PCT groups on substance use outcomes.

		Mean (95% CI)			Mean Difference (95% CI)					
Outcome Measure		Session 1	Session 5	Session 11	Within-Group Difference between Session 1 and Session 5	Between-Group Difference between Session 1 and Session 5	Within-Group Difference Between Session 5 and Session 11	Between-Group Difference Between Session 5 and Session 11	Within-Group Difference Between Session 1 and Session 11	Between-Group Difference Between Session 1 and Session 11
**Quantity Substance Use**	COPE-A	0.81 (0.64 to 0.98)	0.75 (0.53 to 0.97)	0.75 (0.54 to 0.97)	−0.06 (−0.27 to 0.15)	0.02 (−0.22 to 0.26)	−0.09 (−0.33 to 0.14)	−0.19 (−0.57 to 0.20)	−0.15 (−0.36 to 0.05)	−0.17 (−0.49 to 0.16)
	PCT	0.81 (0.67 to 0.96)	0.74 (0.57 to 0.90)	0.73 (0.57 to 0.90)	−0.08 (−0.20 to 0.04)	1 [Reference]	0.09 (−0.21 to 0.40)	1 [Reference]	0.01 (−0.24 to 0.26)	1 [Reference]
	Between-group difference at each interview, Mean (95% CI)	−0.003 (−0.229 to 0.223)	0.02 (−0.25 to 0.29)	−0.17 (−0.25 to 0.29)						
		Mean (95% CI)			IRR (95% CI)					
**Frequency Substance Use**	COPE-A	1.45 (1.20 to 1.70)	1.20 (0.87 to 1.54)	1.00 (0.46 to 1.54)	−0.25 (−0.47 to −0.03)*^a^*	−0.07 (−0.36 to 0.23)	−0.20 (−0.55 to 0.15)	−0.28 (−0.68 to 0.12)	−0.45 (−0.90 to −0.01)*^a^*	−0.34 (−0.86 to 0.17)
	PCT	1.31 (1.06 to 1.57)	1.13 (0.77 to 1.49)	1.21 (0.82 to 1.60)	−0.18 (−0.38 to 0.01)	1 [Reference]	0.07 (−0.11 to 0.26)	1 [Reference]	−0.11 (−0.35 to 0.14)	1 [Reference]
	Between-group difference at each interview, IRR (95% CI)	0.14 (−0.22 to 0.50)	0.07 (−0.43 to 0.56)	−0.21 (−0.88 to 0.46)						

Note. COPE-A = Concurrent Treatment of PTSD and SUD Using Prolonged Exposure, Adolescent version; PCT = Person-Centred Therapy; IRR = incidence rate ratio; CI = confidence interval.

*^a^p* < .05.

## 4. Temporal sequencing of changes

### 4.1. PTSD symptoms

Change scores across individual PTSD symptom clusters within each treatment phase were strongly and positively correlated (*r’s* = .54 to .78; Table 4). In contrast, no significant associations were observed between symptom changes in the early (S1-S5) and later symptom treatment phases (S5-S11); either within the same symptom or between different clusters (*r’s* = –.10 to –.47). However, negative relationships were observed between early and later change in Cluster B (intrusions) symptoms (*r’s* = −.43, *p* = .049) and between early change in Cluster C (avoidance) and later change in Cluster D (negative alterations in cognition and mood) symptoms (*r’s* = −.47, *p* = .030) (Table 5).

**Table 4.** Concurrent changes in DSM-5 PTSD symptom clusters for early and later treatment phases measured with the PTSD-RI.

Change scores			95% CI		
		*r*	*Lower*	*Upper*	*p*
*Early treatment phase (S1 to S5)*					
Cluster B	Cluster C	.65	.46	.92	< .001
Cluster B	Cluster D	.75	.45	.92	< .001
Cluster B	Cluster E	.72	.44	.92	< .001
Cluster C	Cluster D	.68	.20	.87	< .001
Cluster C	Cluster E	.54	.53	.92	< .001
Cluster D	Cluster E	.67	.66	.93	< .001
*Later treatment phase (S5 to S11)*		* *	* *	* *	* *
Cluster B	Cluster C	.74	.34	.93	< .001
Cluster B	Cluster D	.78	.64	.9	< .001
Cluster B	Cluster E	.64	.33	.83	< .001
Cluster C	Cluster D	.62	.32	.83	.002
Cluster C	Cluster E	.70	.38	.9	< .001
Cluster D	Cluster E	.77	.55	.87	< .001

Note. PTSD = posttraumatic stress disorder; PTSD-RI = Posttraumatic Stress Disorder Reaction Index; Cluster B = Intrusions; Cluster C = Avoidance; Cluster D = Negative alterations in cognition and mood; Cluster E = Alterations in arousal and reactivity.

**Table 5.** Temporal changes in DSM-5 PTSD symptom clusters measured with the PTSD-RI.

Change scores			95% CI		
* *	* *	*r*	*Lower*	*Upper*	*p*
*S1-S5*	*S5-11*				
Total PTSD	Total PTSD	−.22	−.69	.22	.335
Cluster B	Cluster B	−.43	−.80	−.04	.049
Cluster C	Cluster C	−.20	−.68	.20	.391
Cluster D	Cluster D	−.22	−.68	.35	.331
Cluster E	Cluster E	−.33	−.75	.09	.148
Cluster B	Cluster C	−.28	−.69	.14	.226
Cluster B	Cluster D	−.26	−.69	.34	.261
Cluster B	Cluster E	−.38	−.79	.14	.092
Cluster C	Cluster B	−.21	−.71	.20	.365
Cluster C	Cluster D	−.21	−.66	.28	.364
Cluster C	Cluster E	−.47	−.84	−.11	.030
Cluster D	Cluster B	−.10	−.68	.38	.667
Cluster D	Cluster E	−0.34	−0.77	.06	.131

Note. PTSD = posttraumatic stress disorder; PTSD-RI = Posttraumatic Stress Disorder Reaction Index; Cluster B = Intrusions; Cluster C = Avoidance; Cluster D = Negative alterations in cognition and mood; Cluster E = Alterations in Arousal and Reactivity.

### 4.2. Substance use symptoms

Changes in the quantity and frequency of substance use within each treatment period were significantly and positively related (*r’s* = .45 and .62; Table 6). No significant temporal associations were observed between early and later changes in substance use outcomes (Table 6); except for a negative association between change in quantity of use from S1 to S5 and quantity between S5 and S11 (*r* = −.52, *p* = .018).

**Table 6.** Concurrent and temporal changes in substance use between early and later treatment phases.

Change scores			95% CI		
* *		*r*	*Lower*	*Upper*	*p*
*Early treatment phase (S1 to S5)*					
Quantity	Frequency	.45	.15	.81	.01
*Later treatment phase (S5 to S11)*					
Quantity	Frequency	.62	.33	.82	.003
*S1-S5*	*S5-11*				
Quantity	Quantity	−.52	−.85	.00	.018
Frequency	Frequency	−.10	−.63	.46	.662
Quantity	Frequency	−.31	−.81	.33	.190
Frequency	Quantity	−.02	−.48	.49	.915

### 4.3. PTSD and substance use symptoms

In regard to concurrent associations, changes in PTSD symptom severity were not significantly associated with changes in either the quantity or frequency of substance use within the same phase of treatment (Table 7). Similarly, no significant temporal associations were found between early changes in PTSD symptoms and later changes in substance use outcomes, or vice versa (Table 7). Across all analyses, correlation patterns did not meaningfully differ when analyses were stratified by intervention group (i.e. COPE-A and PCT).

**Table 7.** Concurrent and temporal changes in PTSD-RI total score and substance use

Change scores			95% CI		
* *		*r*	*Lower*	*Upper*	*p*
*Early treatment phase (S1 to S5)*					
PTSD total	Frequency	−.04	−.45	.56	.802
PTSD total	Quantity	−.10	−.54	.43	.586
*Later treatment phase (S5 to S11)*					
PTSD total	Frequency	.28	−.24	.78	.220
PTSD total	Quantity	.07	−.43	.50	.766
*S1-S5*	*S5-11*				
PTSD total	Quantity	−.06	−.56	.35	.784
PTSD total	Frequency	−.12	−.48	.49	.616
Quantity	PTSD total	.01	−.56	.61	.980
Frequency	PTSD total	.03	−.51	.61	.913

Note. PTSD = posttraumatic stress disorder; PTSD-RI = Posttraumatic Stress Disorder Reaction Index.

## 5. Discussion

There is growing evidence for the efficacy of treatments targeting co-occurring PTSD-SUD, primarily among adults, yet gaps remain in understanding how these symptoms change over time or relate to one another during treatment. This question is particularly salient for youth, where neurodevelopmental processes, social context, and trauma-related vulnerability may shape symptom presentation and treatment response in ways that diverge from adult patterns (Brady & Back, 2012). To our knowledge, the present study is the first to examine within-treatment symptom change among youth, spanning adolescence and emerging adulthood (aged 13–25 years); receiving an integrated, trauma-focused intervention, as well as the temporal associations between PTSD symptom clusters, overall PTSD severity, and substance use across treatment.

Consistent with adult COPE trials compared with relapse-prevention approaches (Back et al., 2019; Ruglass et al., 2017); youth receiving COPE-A showed significantly greater reductions in total PTSD symptom severity compared with PCT. By session 11, mean PTSD scores in the COPE-A group fell below the diagnostic threshold of 35 (Kaplow et al., 2020). Compared with PCT, the COPE-A group demonstrated larger reductions in the specific DSM-5 PTSD symptom clusters B (intrusions); C (avoidance); and D (negative alterations in mood and cognition). Limited between-group differences were observed for Cluster E (alterations in arousal and reactivity; consistent with evidence that this cluster is more resistant to change (Back et al., 2006; Miles et al., 2023)).

Although symptom improvement began early, between-group differences became most pronounced between sessions 5 and 11, coinciding with the initiation of prolonged exposure in COPE-A. This pattern highlights the importance of treatment retention but also suggests that exposure components and trauma-focused skill building may be key drivers of treatment response. This is notable as, despite strong evidence that exposure-based interventions are highly efficacious in reducing PTSD symptoms including among individuals with comorbid SUD (Hien et al., 2024); many clinicians remain reluctant to deliver exposure components, citing concerns about tolerability, safety, and ethics (Borah et al., 2017; Deacon et al., 2013; Meyer et al., 2014; Murray et al., 2022). Together with research questioning the necessity of phase-based approaches that delay exposure until later stages of treatment and may prolong care without added benefit (Oprel et al., 2021; van Vliet et al., 2021); our findings support the early introduction of exposure-based components as critical to PTSD symptom change.

Both COPE-A and PCT produced comparable trajectories of substance-use outcomes, with no significant between-group differences in either quantity or frequency of use. However, participants in COPE-A demonstrated modest within-group reductions in substance-use frequency over time, while no significant changes were observed for PCT. These findings may indicate that trauma-focused treatment facilitates secondary improvements in substance use, even when overall reductions are not significantly greater than those observed with supportive-counselling care. These findings have two important implications. First, they suggest that integrated exposure-based approaches are efficacious for trauma symptoms and can be delivered to youth across adolescence and early adulthood in the context of ongoing substance use. Second, the reductions in PTSD symptoms and other forms of non-SUD psychiatric comorbidity found for COPE-A here and in the overall trial (Mills et al., 2020); represents a meaningful reduction in the overall allostatic load, or burden of having symptoms of multiple disorders, on the individual. Nevertheless, additional or tailored supports, such as adjunctive pharmacotherapies (Hien et al., 2024); may be required to promote further improvements in substance use.

These findings may also highlight that frequency and quantity represent related but distinct dimensions of substance use, which may change with differing magnitude across treatment. There has been limited research examining differential treatment effects on quantity versus frequency of substance use, particularly in youth; however, some prior work has reported greater reductions in frequency relative to quantity following substance-use interventions (Martin & Copeland, 2008). Interpretation of quantity outcomes in the present study is constrained by measurement and analytic challenges, including the need to standardise quantity across substances and to transform values (e.g. log-transformation and standardisation) to meet distributional assumptions. As a result, changes in transformed quantity metrics may be less interpretable than frequency-based outcomes. Accordingly, these findings should be interpreted cautiously, particularly given the modest sample size, but nonetheless underscore the potential clinical value of monitoring both frequency and quantity of substance use across treatment.

To elucidate the temporal sequencing of symptom change during treatment, we examined concurrent and temporal relationships between changes in PTSD and substance use outcomes. This is the first study in nearly two decades (Back et al., 2006) to examine the temporal relationship between early and late treatment change in PTSD symptoms and substance use symptoms. For PTSD, changes in DSM-5 PTSD symptom Cluster (B: intrusions, C: avoidance, D: negative alterations in mood and cognition, and E: alterations in arousal and reactivity) were strongly correlated within each treatment phase, indicating that these symptom improvements tended to co-occur within the same treatment phases, rather than following a clearly sequential pattern across phases. Unlike prior adult work suggesting that changes in Cluster B and E earlier in treatment may precede later improvements in other clusters (Back et al., 2006); we found no evidence that early change in any cluster predicted subsequent change in others. These findings suggest that, among youth with PTSD-SUD, PTSD symptoms may improve holistically across treatment, although the use of aggregated change scores limits inference regarding more fine-grained temporal ordering and further research using more analytically robust methods is required to confirm these findings.

Substance use outcomes showed a similar pattern: quantity and frequency were positively correlated within each treatment phase, with limited temporal relationships between phases. One exception was a moderate negative association between early and later change in substance use quantity. Consistent with these patterns, we observed no significant concurrent or lagged associations between changes in PTSD and substance use. Early PTSD improvement did not predict later reductions in substance use, nor did early substance use change predict subsequent PTSD improvement. This lack of observed association suggests that, within the limits of the current analytic approach and sample size, changes in PTSD symptoms were not temporally coupled with changes in substance use during treatment. While further research is needed to confirm if changes in PTSD and substance-use symptoms occur through independent mechanisms, these findings may highlight the importance of addressing both conditions when working with individuals in this developmental period. While our findings align with previous studies among adults reporting no associations between PTSD and substance use symptom change during treatment (Hien et al., 2025; Badour et al., 2022; Peirce et al., 2020); they contrast with other work reporting that PTSD reductions precede or mediate declines in substance use or craving (Back et al., 2006; Hien et al., 2025; Tripp et al., 2020; Hien et al., 2018; Kaczkurkin et al., 2016; Ouimette et al., 2010).

Several factors may explain these divergent findings. In addition to differences in sample characteristics (e.g. adult or veteran populations); prior research has often focused on alcohol use specifically, whereas our sample comprised youth with diverse substance-use profiles. Second, weekly assessments may not have captured more distal or cumulative effects; a recent meta-analysis of integrated treatments found substance use improvements frequently emerged more than five months post-intervention (Roberts et al., 2016). Third, differences in intervention design and analysis may account for discrepancies, for example, some studies incorporated pharmacotherapy (Back et al., 2006; Kaczkurkin et al., 2016); examined substance cravings rather than use (Kaczkurkin et al., 2016); and defined categorical response groups within their analysis (e.g. only treatment responders) (Back et al., 2006; Hien et al., 2010). Trials also varied widely in duration (6–26 weeks) and in measurement method (self-report vs clinician-rated).

This study has several strengths. To our knowledge, it is the first to examine within-treatment changes in both PTSD and substance use symptoms among youth receiving integrated care, extending previous work in adults and informing early intervention approaches in this developmentally vulnerable population. Participants were drawn from a treatment-seeking sample with high clinical complexity (Peach et al., 2024); enhancing ecological validity. Weekly assessments enabled fine-grained analysis of symptom trajectories, and separate assessment of both the frequency and quantity of substance use addressed a key limitation of earlier research, which has relied on only frequency or abstinence-based outcomes (Hien et al., 2018).

Despite these strengths, several limitations should be noted. First, standard GEE inference relies on large-sample properties in the number of independent clusters (Gunsolley et al., 1995; Liang & Zeger, 1986). Given the small number of independent clusters at later sessions, robust standard errors were estimated using a bias-adjusted covariance estimator to improve small-sample properties (Mancl & DeRouen, 2001). However, results should be interpreted cautiously. In particular, tests of group × time interactions are likely to be underpowered, which may limit the ability to detect differential treatment effects even when within-group changes are observed. This is illustrated by outcomes such as PTSD Cluster E symptoms and substance use frequency, where significant within-group improvements were observed in COPE-A, but corresponding interaction effects were not detected.

Second, although attrition is common in PTSD-SUD integrated treatment trials (Roberts et al., 2016); the modest sample size constrained the use of data from all treatment sessions (i.e. up to 16) and precluded more sophisticated longitudinal methods (e.g. cross-lagged panel or session-level lagged models) to examine temporal sequencing. To mitigate the risk of unstable estimates and overfitting under these conditions, phase-aggregated change scores were used as a pragmatic alternative for exploratory examination of within-treatment processes. Small group sizes also limited the ability to examine temporal sequencing separately by treatment condition; however, supplementary analyses suggested that correlation patterns did not differ meaningfully when stratified by intervention group, consistent with previous research (Hien et al., 2010; Tripp et al., 2020). These limitations underscore the need for larger, methodologically rigorous studies that apply session-level lagged or latent change models to more rigorously evaluate temporal sequencing and mechanisms of change in youth with PTSD-SUD.

Third, reliance on retrospective self-report outcomes may have introduced recall inaccuracies and social desirability bias, and findings may be partly dependent on the specific PTSD and SUD outcome measures used in the trial from which the present data were drawn. Finally, although focusing on participants’ primary substance of concern aligned outcomes with treatment goals, this approach may have obscured changes in secondary substance use throughout treatment. Future studies should more comprehensively examine patterns of polysubstance change during treatment to determine whether alternative trajectories or temporal relationships emerge.

Developmental heterogeneity is also an important consideration**.** The sample spanned both adolescents (defined as aged 12–17 years, although all participants in this sample were 13 years of age and older) and emerging adults (aged 18–25 years); who occupy distinct developmental stages with differing social, legal, and contextual demands that may influence trauma-related symptoms, substance use, and their interaction (Hall et al., 2016; Gilhooly et al., 2018; Tucker et al., 2021; Tanner & Arnett, 2016). At the same time, contemporary developmental and life-course research increasingly recognises the transition from adolescence to adulthood as extended and asynchronous, particularly in high-income settings, with biological maturation occurring earlier while social and economic transitions are delayed into the early to mid-twenties (Skirbekk et al., 2025; Sawyer et al., 2018; Twenge & Park, 2019).

Nevertheless, the present study was not powered to examine developmental moderators of within-treatment change. Although adolescents and emerging adults were analysed together, prior analyses of this trial found limited differences in baseline clinical characteristics between these age groups across relevant domains (Peach et al., 2024); supporting cohort-level analysis while acknowledging that age-specific mechanisms may exist. Importantly, the modest sample size limited the ability to disentangle developmental or contextual influences on symptom change, including factors such as legal access to alcohol at age 18 in Australia, which may have shaped substance use trajectories independently of treatment effect. Future research should examine whether developmental period moderates frequency and quantity trajectories and other treatment processes during integrated PTSD-SUD care.

In summary, this study supports integrated, trauma-focused interventions for youth with comorbid PTSD-SUD. Exposure-based treatment produced meaningful reductions in PTSD symptoms, while substance use also decreased during treatment, though not significantly relative to control. Improvement in both PTSD and SUD severity reinforces the safety and tolerability of prolonged exposure in youth with severe comorbidity, and challenges longstanding concerns that therapist-led exposure may exacerbate substance use. However, PTSD improvements did not appear to directly translate into significant reductions in substance use, highlighting the potential need for adjunctive or extended supports. Examination of temporal patterns offers insight into when treatment effects occur, and which treatment components may be most effective. These findings refine theoretical models of PTSD-SUD comorbidity and inform strategies to optimise treatment delivery. Effective intervention during these developmental periods may lessen the long-term burden of co-occurring PTSD-SUD.

## Data Availability

Due to the nature of the research, supporting data is not available to protect the confidentiality of the participants.
